# The UmuC subunit of the *E. coli* DNA polymerase V shows a unique interaction with the β-clamp processivity factor

**DOI:** 10.1186/1472-6807-13-12

**Published:** 2013-07-04

**Authors:** Atif A Patoli, Jody A Winter, Karen A Bunting

**Affiliations:** 1 Centre for Genetics and Genomics, University of Nottingham, Queen’s Medical Centre, Nottingham NG7 2UH, UK; 2 Current address - Centre for Biomolecular Sciences, University of Nottingham, University Park, Nottingham NG7 2RD, UK; 3 Current address – Novozymes Biopharma UK, Castle Court, 59 Castle Boulevard, Nottingham NG7 1FD, UK

**Keywords:** Translesion synthesis, Sliding clamp, Processivity factor, UmuC, Regulation

## Abstract

**Background:**

Strict regulation of replisome components is essential to ensure the accurate transmission of the genome to the next generation. The sliding clamp processivity factors play a central role in this regulation, interacting with both DNA polymerases and multiple DNA processing and repair proteins. Clamp binding partners share a common peptide binding motif, the nature of which is essentially conserved from phage through to humans. Given the degree of conservation of these motifs, much research effort has focussed on understanding how the temporal and spatial regulation of multiple clamp binding partners is managed. The bacterial sliding clamps have come under scrutiny as potential targets for rational drug design and comprehensive understanding of the structural basis of their interactions is crucial for success.

**Results:**

In this study we describe the crystal structure of a complex of the *E. coli* β-clamp with a 12-mer peptide from the UmuC protein. UmuC is the catalytic subunit of the translesion DNA polymerase, Pol V (UmuD’_2_C). Due to its potentially mutagenic action, Pol V is tightly regulated in the cell to limit access to the replication fork. Atypically for the translesion polymerases, both bacterial and eukaryotic, Pol V is heterotrimeric and its β-clamp binding motif (^357^ QLNLF ^361^) is internal to the protein, rather than at the more usual C-terminal position. Our structure shows that the UmuC peptide follows the overall disposition of previously characterised structures with respect to the highly conserved glutamine residue. Despite good agreement with the consensus β-clamp binding motif, distinct variation is shown within the hydrophobic binding pocket. While UmuC Leu-360 interacts as noted in other structures, Phe-361 does not penetrate the pocket at all, sitting above the surface.

**Conclusion:**

Although the β-clamp binding motif of UmuC conforms to the consensus sequence, variation in its mode of clamp binding is observed compared to related structures, presumably dictated by the proximal aspartate residues that act as linker to the poorly characterised, unique C-terminal domain of UmuC. Additionally, interactions between Asn-359 of UmuC and Arg-152 on the clamp surface may compensate for the reduced interaction of Phe-361.

## Background

Temporal and spatial regulation of events during DNA replication and repair is crucial to the maintenance of genome stability and accurate transmission of the genome to the next generation. Protein-protein interactions are central to such regulation within the *E. coli* replisome and characterisation of these interactions is critical to our understanding of these complex, dynamic processes [1]. Processivity factors play an integral role involving both protein-protein and protein-DNA interactions in regulation of events at the replication fork [2,3].

Processivity factors, or sliding clamps, such as the prokaryotic β-clamp and eukaryotic and archaeal proliferating cell nuclear antigen (PCNA), show a high degree of structural conservation despite limited sequence identity [4]. These ring–shaped proteins topologically encircle DNA providing a sliding platform for the majority of DNA polymerases and many DNA-interacting proteins. Most typically these partners bind to the processivity factor via a conserved motif (β-clamp: QL[S/D]LF and PCNA: Qxx[I/L/M]xxF[F/Y]) at their extreme N- or C-terminus, although internal motifs are not unknown [5,6]. Structures of binding partners in complex with processivity factors have demonstrated that a variety of modulating interfaces exist beyond this principal motif [7-9]. Given the number of postulated binding partners of sliding clamps such interfaces are clearly important in adding levels of subtlety to processivity factor access and the establishment of binding hierarchies [10].

The involvement of processivity factors in the regulation of the Y-family of translesion polymerases has attracted much research interest over the last decade. These polymerases are potentially mutagenic and as such their access to the primer-template terminus must be strictly regulated. The principal interaction motifs have been widely characterised structurally as well as by protein complexes, showing modulating, regulatory interfaces [7,11-13]. Four of the five known *E. coli* polymerases, including the translesion polymerases IV and V, are known to require functional interaction with the β-clamp for *in vivo* activity and to date structures of the clamp-binding peptides of Pol II, Pol III and Pol IV have been solved in complex with the β-clamp leaving only the UmuC motif uncharacterised [11,14].

The bacterial β-clamps have attracted much interest as potential targets for antibiotic therapy since all five DNA polymerases interact with the same site on the clamp [15] and critically, inhibitors of prokaryotic polymerase binding do not inhibit eukaryotic PCNA-binding partner interactions [14]. Rational design of inhibitors of this binding pocket could lead to development of broad spectrum and species-specific antibiotics and it is of great importance to thoroughly characterise binding pocket interactions, since inhibitors can differentially affect the various *E. coli* DNA polymerases [14].

**Figure 1:**
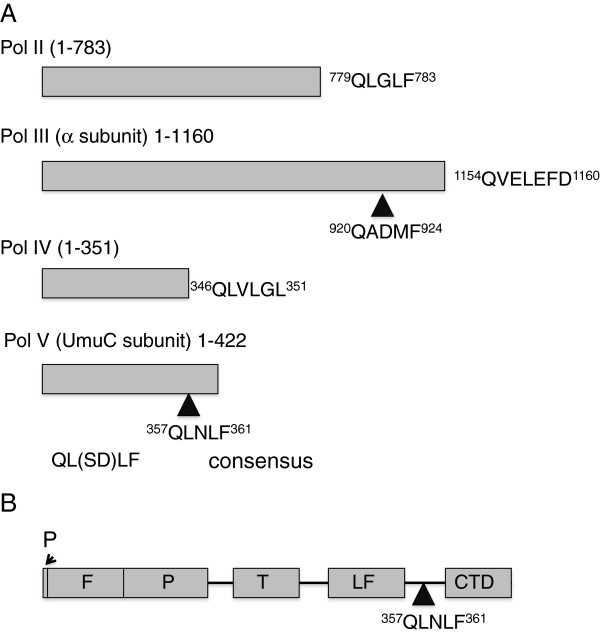
**The β-clamp binding motifs. A**. Schematic to show the relative positions and sequences of the β-clamp binding motifs in Pol II, Pol III, Pol IV and Pol V. Shown for comparison is the consensus β-binding motif as identified by Dalrymple and others (2001) [5]. **B**. A more detailed schematic of Pol V showing the β-binding motif in relation to known domains. Domains: F – finger; P – palm, T – thumb, LF – little finger and CTD – C-terminal domain.

Pol V is a heterotrimer consisting of the catalytic UmuC subunit and a homodimer of UmuD’ and was originally identified as being central to damage-induced mutagenesis. Given this mutagenic potential it is subject to many levels of regulation [16]. It is transcriptionally regulated as part of the *E. coli* SOS response and undergoes further post-translational modification whereby the UmuD subunit undergoes RecA-stimulated auto-catalytic cleavage to produce the functional UmuD’_2_C heterotrimer. A role for UmuD is emerging in the regulation of mutagenesis and in control of polymerase access to the sliding clamp [17,18]. The β-clamp-UmuC interaction provides a further level of regulation and Dalrymple and others identified an internal β-binding motif ^357^QLNLF^361^, located on the linker region between the little finger (LF) domain and the C-terminal domain (CTD) of UmuC [5]. Although the translesion polymerases share the overall right hand fold of classical polymerases, comprising a palm, fingers and thumb domain, the family possesses a unique LF domain crucial to lesion bypass. In Pol IV, the β-binding motif is proximal to the LF and the β-binding motif of UmuC motif is in an analogous position (Figure 1). The CTD of UmuC is of unknown function and is presumably a later acquisition. Pol V is the only family member to have accessory subunits in the form of the UmuD’_2_ homodimer. Analysis of UmuC mutants demonstrated the functional importance of the CTD in Pol V and it has been suggested that the UmuC CTD mediates the interaction between UmuC and UmuD’_2_ in the Pol V heterotrimer [19].

Internal β-binding and PCNA-interacting peptide (PIP) boxes are less common than those located at the extreme termini but they are by no means unknown. The main *E. coli* replicase Pol III catalytic subunit has been postulated to possess two β-binding motifs, one internal and one at the extreme C-terminus, although there is some debate as to the relative roles of the two sites, with the C-terminal peptide solved in complex with the β-clamp [14,20,21]. The peptide-binding pocket of the β-clamp has been proposed as a target for rational drug design to disrupt bacterial growth and a candidate molecule co-crystallised with the clamp [14,22]. We wished to complete the structural analysis of the *E. coli* DNA polymerase β-binding motifs by solving the UmuC clamp-binding peptide in complex with the β-clamp.

## Methods

### Expression and purification of β-clamp

Plasmid pACYC11-*dnaN* encoding full length β-clamp with an N-terminal His-tag was transformed into *E. coli* B834 (DE3) expression strain [23]. Fresh transformants were grown in LB broth containing 34 μg/ml chloramphenicol at 37°C. Cells were induced with IPTG (0.1 mM final concentration) at OD_600_ = 0.6-0.8, and were further incubated overnight at 25°C. The cells were then harvested by centrifugation, lysed by sonication in buffer A (50 mM HEPES pH 7.0, 200 mM NaCl, 20 mM imidazole) and the soluble fraction obtained by centrifugation at 15,000 *x g* for 30 min at 4°C. The soluble fraction was then applied to a batch Talon affinity column (Clontech) equilibrated in buffer A, washed with 30 column volumes of buffer A and eluted in a small volume of buffer A supplemented to 300 mM imidazole. The protein was then applied directly to a 26/60 Superdex 200 column equilibrated and run in 50 mM HEPES pH 7.0 and 200 mM NaCl. Fractions containing purified β-clamp were pooled and concentrated to 25 mg/ml.

### Dylight649-Pol V synthetic peptide

Purified peptide (^352^QGVAQLNLFDD^363^) corresponding to the β-clamp binding region of UmuC was linked with Dylight649 chromophore at the N-terminus (Cambridge Peptides). A cysteine bridge was included to permit linkage of the chromophore to the peptide i.e. (Dylight649-C-^352^QGVAQLNLFDD^363^). The peptide was suspended in 50 mM HEPES, 200 mM NaCl to a final concentration of 1 mM.

### Purification and crystallization of β-clamp in complex with Dylight649-Pol V synthetic peptide

Purified β-clamp, at a concentration of 0.6 mM was mixed with an excess of the Dylight649-UmuC synthetic peptide, at 1 mM. After 1 hour incubation at room temperature, the clamp-peptide complex was purified by size-exclusion chromatography (10/300 Superdex 200 column) and concentrated to 15 mg/ml. The complex was crystallized in a solution containing 200 mM calcium acetate, 200 mM MES pH 6.5, 14% PEG 6000 using the sitting drop method at 12°C, in a 1:1 ratio with the well solution. Crystals were cryo-protected by transfer to equilibrated well solution supplemented with 15% PEG 400 prior to freezing.

### Structure solution

Data were collected at Diamond Light Source (IO2) and processed using iMOSFLM and the CCP4 suite [24,25]. Molecular replacement was performed using Balbes, producing a clear solution utilising [PDB:3D1G] as a search model [14,26]. Clear density was visible in a *Fo-Fc* map in the expected location of the UmuC peptides. However, to avoid bias, initial building of the peptide backbones was performed using Buccaneer, followed by rounds of manual model building using Coot and refinement using Refmac 5, interspersed with monitoring using Molprobity [27-30]. Tight NCS restraints were employed during refinement, excluding regions where inspection of maps suggested deviation between the subunits. Initial TLS parameters for refinement were calculated using the TLS Motion Determination Server (http://skuld.bmsc.washington.edu/~tlsmd/). Coordinates have been deposited with the PDB [PDB:4K74].

## Results

### Global architecture

**Figure 2:**
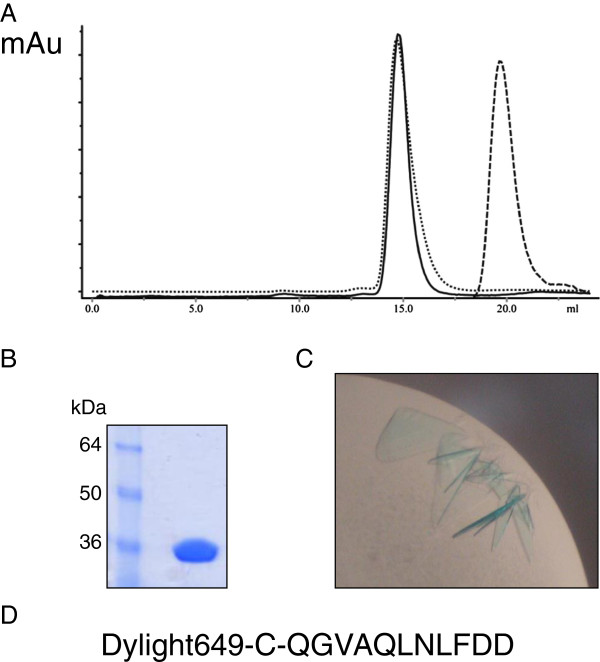
**Production of β-clamp/UmuC peptide co-crystals. A**. Size exclusion traces of β-clamp protein (unbroken), UmuC peptide (dashed) and complex (dotted), showing the expected shift in elution volume on complexation. *x* axis – elution volume (ml), *y* axis absorption OD280. **B**. SDS-PAGE analysis of His-tagged β-clamp. **C**. β-clamp/UmuC co-crystals showing the pronounced blue colouration from the Dylight649 chromophore. **D**. Peptide sequence.

The structure of the *E. coli* β-clamp was solved in complex with a fluorophore-labelled synthetic UmuC β-binding peptide to 2.5 Å resolution (Table 1). Utilisation of a labelled peptide, as described previously by Georgescu and others [14], permitted the ready identification of protein-peptide complex in the crystallisation trays due to a marked blue colouration of the crystals (Figure 2), over-coming a significant impediment to solution of β-clamp complexes due to the fact that unbound clamp protein crystallises very readily. Analysis of the complex via size exclusion chromatography (Figure 2) showed the expected slight reduction in elution volume, consistent with a small increase in size of the clamp-peptide complex versus the clamp alone. The resulting fractions were bright blue in colour, again consistent with the formation of a complex between the clamp protein and UmuC peptide.

**Table 1:** Data collection and refinement statistics

	**All data (outer shell)**
**Data collection**	
Space group	P2_1_
Cell dimensions	
a,b,c (Å)	79.5, 66.2, 82.7
α,β,γ (°)	90.0, 115.0, 90.0
Resolution (Å)	72.07-2.50 (2.64-2.50)
R_merge_	0.062 (0.284)
I/σI	7.7 (2.5)
Completeness (%)	92.1 (93.6)
Redundancy	1.9 (1.9)
**Refinement**	
Resolution (Å)	70.00-2.50
No. of unique reflections	22294
R_cryst_	23.2
R_free_	29.5
Number of atoms/au	5726
Rms deviation from ideal values:	
Bond lengths (Å)	0.01
Bond angles (°)	1.37
Average temperature factors:	
β-clamp (chain A/B)	21.6/21.8
Peptide (chain C/D)	27.2/26.3
Water molecules (W)	25.4

The asymmetric unit contains a single β-clamp dimer with a peptide bound to each monomer. Due to insufficient electron density to confidently model loop regions, β-clamp Chain A residues 22–26 and 209–211 and Chain B residues 22–23 were excluded during model building. The terminal residue of the hexa-histidine tag has been modelled in both subunits, with the remainder of the tag presumably disordered. The C-terminal residues (Arg-365 and Leu-366) in both β-clamp chains (A and B) were too disordered to permit modelling with any degree of confidence, as were the side chains of Arg-73 (A), His-148 (A), Arg-100 (B) and Phe-230 (B). The 12-mer peptide consisted of Dylight649-C-QGVAQLNLFDD^363^, with a cysteine included to permit linkage of the fluorophore to the peptide. The density for peptide chain D (associated with β-clamp chain B) (Additional file 1: Figure S1) was better defined than peptide chain C (associated with β-clamp chain A). Variations in occupancy have previously been observed in β-clamp-peptide complexes, with one site being occluded by crystal packing [11,14]. Residues ^356^AQLNLF^361^ were modelled for chain D and ^357^QLNLF^361^ for chain C, comprising the core β-binding motif of the UmuC peptide, with the remaining residues presumably disordered. NCS restraints were not applied to the terminal modelled residue for the peptide, Phe-361, since inspection of the *2F*_*o*_*-F*_*c*_ and *F*_*o*_*-F*_*c*_ maps during refinement suggested variation for this residue between the two peptide chains. Areas of positive density surrounding the modelled residue in chain C are suggestive of some variation in position of the side chain but were not sufficiently well-defined to support inclusion of alternate conformations within the model. Aside from this residue the two peptide chains are very similar. The disposition of secondary structural elements in the β/UmuC peptide complex is retained between the uncomplexed β-clamp structure [PDB:2POL] and β-clamp complexes with clamp-binding peptides from Pols II [PDB:3D1E], III [PDB:3D1F] and IV [PDB:1OK7]. For reference, key interacting residues from each of these complexes are mapped in Additional file 1: Figure S2. Amino acid numbers given will refer to chains B (β-clamp) and D (UmuC peptide) of the complex, unless otherwise stated.

### Interaction of the UmuC peptide with β-clamp

**Figure 3:**
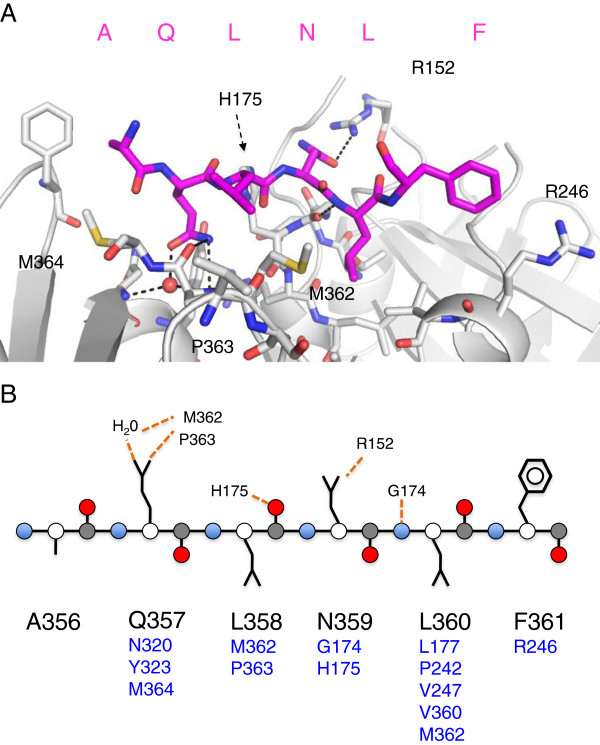
**Interactions between the UmuC peptide and β-clamp. A**. The clamp is coloured in gray and the peptide in magenta/atomic colouring. Interacting residues are depicted in stick representation with hydrogen bonds shown as black dotted lines. Key interacting residues from the β-clamp are labelled. **B**. Schematic of the peptide/clamp interactions. Backbone atoms are indicated by circles: Cα (white), C (gray), O (red) and N (blue) with side chains in line representation. Hydrogen bonds are indicated by dashed orange line with the indicated β-clamp residues. Proximal clamp residues are shown in blue under the respective peptide residue.

Unlike the neighbouring Ala-356, only ordered in chain D, peptide Gln-357 forms intimate interactions with the clamp surface as seen in the other peptide-clamp complexes (Figure 3). The OE1 group forms a hydrogen bond with a conserved solvent molecule, with the NE2 group forming two hydrogen bonds with the main chain carbonyls of Met-362 and Pro-363 on the clamp. The carbonyl group of Met-362 also forms a hydrogen bond with the solvent molecule. Peptide Leu-358 does not make any contacts with the clamp surface and is oriented towards the solvent. The density for peptide Asn-359 is well-defined, due to a hydrogen bond formed between its OD1 group and the NH2 group of Arg-152 on the clamp surface. Peptide Leu-360 is located within the hydrophobic pocket on the clamp surface defined by residues Val-247, Leu-177, Val-360, Pro-242, Met-362, with the main chain nitrogen forming a hydrogen bond with the carbonyl group of Gly-174.

**Figure 4:**
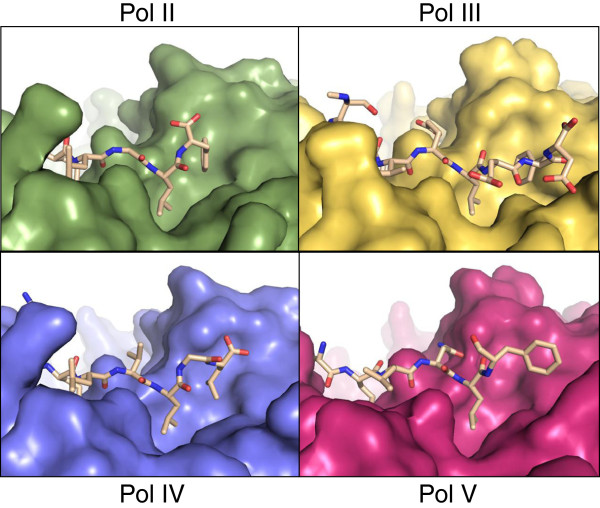
**Deviations are observed in the disposition of the β-binding motif.** The clamps are shown in surface form and the peptides in stick representation, with atomic colouring. Complexes shown are: **Pol II** [PDB:3D1E], **Pol III** [PDB:3D1F], **Pol IV** [PDB:1UNN] and **Pol V**/UmuC [this study] [7,14]. Individual figures derive from superimposed structures to yield a view of the entire binding pocket in the same orientation in each instance, with the N-terminus of the peptides to the left of the view and the C-terminus to the right.

Remarkably, and in contrast to the other peptide-clamp complexes, peptide Phe-361 does not penetrate the hydrophobic pocket of the clamp and is solvent exposed (Figure 4). The principle interaction appears to be hydrophobic in nature, with the hydrophobic portion of the clamp Arg-246 side chain. Inspection of the hydrophobic pocket on the β-clamp typically occupied by the peptide aromatic component suggested that the pocket is empty in this complex. The terminal two residues of the peptide, both aspartate, appear to be disordered. In the context of the intact UmuC protein, they would function as a linker between the LF domain/β-binding motif and the CTD. Since the mode of interaction, if any, of the UmuC CTD with the β-clamp is not known, the possibility cannot be excluded that these residues might provide a more intimate association in the full length complex.

### Comparison of β/UmuC peptide with the uncomplexed β-clamp

The terminal two residues of the β-clamp (Arg-365 and Leu-366) are modelled in the uncomplexed structure, but are not ordered in the peptide-clamp complex. Although there is a clear shift in the position of the clamp Met-362 side chain, noted in other complexes [11], no variation is seen in the disposition of the remaining residues comprising the hydrophobic cleft on the surface of the clamp (Additional file 1: Figure S3). The Arg-152 guanidinium group shifts slightly in position, with the remainder of the side chain in the same orientation and clamp Arg-246 shows variation in its side chain orientation and is proximal to the peptide Phe361. These differences in the disposition of clamp Arg-152 and Arg-246 likely occur on complexation to the UmuC peptide following interactions with Asn-359 and Phe-361 of UmuC, respectively.

### Comparison with Pol II peptide

**Figure 5:**
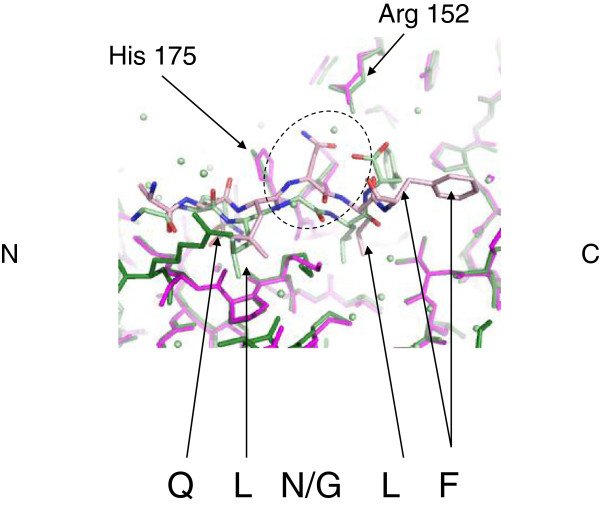
**Comparison of the UmuC peptide with the Pol II peptide.** Only a single amino acid change in observed between the β-binding motifs. The structures are shown in stick representation with atomic colouring, solvent molecules are displayed as spheres. The UmuC complex is shown with the clamp residues and associated solvent molecules in magenta and the peptide in light pink. The Pol II complex is shown with the clamp residues in green and the peptide and solvent molecules in light green. Positions of the binding motif are indicated by arrows where visible in the view. The Gly-781/Asn-359 substitution is highlighted by a dashed circle, indicating the presence of a solvent molecule in the Pol II structure replacing the asparagine side chain from the UmuC peptide. The positions of Arg-152 are identical in the two structures. The N- and C-terminal regions of the peptide are indicated.

Pol II peptide (TLMTGQLGLF) was only found to bind one subunit of the β-clamp, with crystal contacts occluding the other binding pocket [14]. As seen in UmuC, Pol II possesses a 5-amino acid binding motif. The Pol II motif is very similar to that found in UmuC except for glycine replacing asparagine at position 359 (UmuC numbering, unless otherwise stated) (Figure 1, Additional file 1: Figure S2). Some deviation is observed in the refined position of the conserved glutamine side chain of the Pol II clamp binding motif, but this is still oriented such that it can make equivalent hydrogen bonds to the conserved water molecule and the carbonyl oxygen of Met-362 on the clamp (Figure 5). The orientation of the clamp His-175 side chain is altered between the two structures, extending the distance between the peptide Leu-358 carbonyl group and His-175 ND1 from 3.3 (β-clamp-Pol II peptide) to 3.5 Å (β-clamp-UmuC peptide).

The Pol II peptide contains glycine at the equivalent position to UmuC Asn-359 and a solvent molecule is present 1.07 Å from OD1 of the UmuC asparagine. This solvent molecule forms a rather short hydrogen bond with NH2 of clamp Arg-152 (2.62 Å), with the side chain in a virtually identical in position in the two structures (Figure 5). The solvent molecule also interacts with the terminal oxygen of the Pol II peptide, absent in the UmuC peptide.

The Pol II leucine residue equivalent to UmuC Leu-360 is more intimately associated with the β-clamp but shows a similar disposition and both UmuC Leu-360 and the Pol II equivalent are located within the clamp hydrophobic pocket. The biggest variation is seen in the terminal residue of each clamp-binding motif, conserved in each case as a bulky aromatic residue. In the Pol II structure the phenylalanine fits firmly into the hydrophobic pocket on the clamp surface, whilst in UmuC it projects away from the surface of the clamp (Figures 4 and 5), however calculations of the β-clamp surface area involved in the interfaces between the two peptides are comparable. There is a progressive deviation in the main chain position from the conserved glutamine towards the phenylalanine (Table 2), which is more pronounced than variations between major β-clamp residues at the protein-peptide interface.

**Table 2:** Cα to Cα distance (Å) between UmuC-peptide complex structures and previously solved complexes

**Residue - peptide**	**Pol II**	**Pol III**	**Pol IV**
**Ala**/Gly(II)/Glu(III)/Arg(IV)	0.84	0.79	0.67
**Gln**	0.74	0.39	0.73
**Leu/**Val(III)	1.43	1.65	0.78
**Asn**/Gly(II)/Glu(III)Val(IV)	1.39	1.15	0.95
**Leu**	1.51	1.46	1.00
Phe/Gly(IV)	2.90	n/a (insertion Glu)	2.31
Residue – β-clamp	Chain A (peptide associated)	Chain B	Chain B
D150	0.05	0.82	1.06
R152	0.18	0.81	1.05
G174	0.41	0.45	0.42
H175	0.51	0.87	1.12

The UmuC residue is indicated in bold type. For β-clamp comparisons chain B was employed in measurement and compared with the designated chain.

### Comparison with Pol III peptide complex

The sequence of the C-terminal Pol III β-binding motif (QVELEFD) is more distant from the canonical sequence defined by Dalrymple and others (QL[S/D]LF) [5]. The conserved glutamine forms similar interactions to those seen in other complex structures, with the conserved water molecule and the backbone carbonyls of Met-362 and Pro-363 on the clamp. The Pol III valine equivalent to UmuC Leu-358 shows a large shift in the Cα position, resulting in the main chain nitrogen interacting with the carbonyl group of clamp Pro-363. The two side chains are similarly oriented.

**Figure 6:**
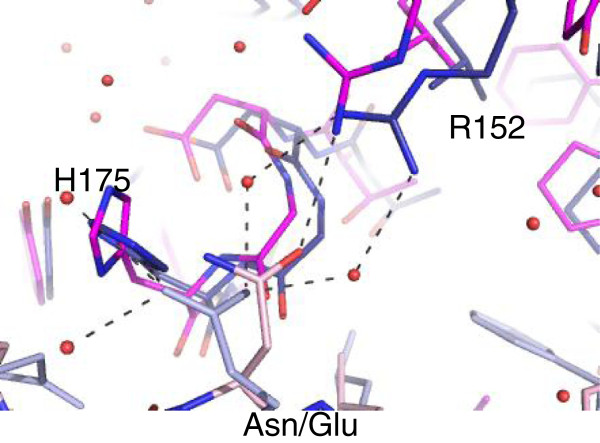
**View of the hydrogen bonding pattern surrounding Asn-359.** Asn-359 in UmuC (magenta) and Glu-1156 in the Pol III peptide (blue) are shown. The glutamate side chain in Pol III forms hydrogen bonds with a network of solvent molecules (all shown molecules relate to Pol III), which in turn interact with Arg-152 and, potentially, the side chain of His-175, resulting in a change in orientation of this residue. Asn-359 forms a hydrogen bond with Arg-152 in Pol V altering its position relative to the Pol III structure.

The Pol III glutamate at the equivalent position to UmuC Asn-359 forms a number of hydrogen bonds with solvent molecules (Figure 6) and with the side chain of His-175 on the clamp (observed in both peptide chains if the side chain of His-175 in Chain B undergoes a 180° rotation) resulting in a change in orientation of this side chain. Two of the solvent molecules form additional hydrogen bonds with the clamp Arg-152 guanidyl group, again affecting the orientation of this side chain compared to that seen in UmuC.

The nitrogen of the Pol III leucine equivalent to UmuC Leu-360 forms a main chain hydrogen bond to the carbonyl group of clamp Gly-174, as seen with UmuC. Possessing a 6-amino acid motif, Pol III contains an additional surface exposed glutamate residue prior to the conserved phenylalanine. The phenylalanine, as in Pol II, is oriented to sit in the hydrophobic pocket on the clamp surface.

Interestingly, both Pol III and UmuC clamp-binding motifs contain aspartate residues C-terminal to the conserved phenylalanine residue. This aspartate is absent from the β-clamp-UmuC peptide structure but is modelled in the Pol III structure, with its OD1 group forming a hydrogen bond with clamp Arg-246 (NH1). This aspartate appears to be further stabilised by hydrogen bonds with the neighbouring phenylalanine carbonyl oxygen via its main chain nitrogen and OD2 groups.

### Comparison with Pol IV structures

The Pol IV β-binding motif deviates more sharply from the canonical sequence (QLVLGL), with a glycine allowing the peptide to bend and insert the flanking leucines into the hydrophobic pocket to mimic the action of the bulkier aromatic residues typically found in this position [7]. The backbone of the Pol IV peptide most closely follows that of UmuC peptide up to the glycine equivalent to Phe-361 (Table 2), again with the UmuC Phe-361 sitting more exposed than the Pol IV LGL motif that mimics the conserved phenylalanine in the clamp’s hydrophobic binding pocket. Aside from this deviation, only subtle differences are noted; the side chain of clamp His-175 is rotated around, presumably due to the lack of a hydrogen bond to the main chain carbonyl of peptide Leu-358, seen in the UmuC structure. As in the other comparative structures the extreme C-terminal residue of Pol IV is represented in the peptide and is ordered, in direct contrast to the internal motif of UmuC, interacting with clamp Arg-152 via solvent molecules.

## Discussion

The nature of the interaction between processivity factors and their binding partners is essentially conserved from phage through to humans [7]. The interaction involves two subsites on the surface [11]. Subsite 1 consists of a hydrophobic pocket on the surface of the processivity factor into which bacterial partners insert the Leu-Phe motif in an extended context, with PIP proteins inserting Phe-Phe involving a loop of 3_10_ helix. Subsite 2 involves interactions of the conserved glutamine residue with solvent molecules and the backbone of the C-terminal region of the clamps.

Given both the central role of the processivity factors in DNA replication and repair and the sheer number of binding partners with highly similar interaction motifs, much attention has been given to how organisms coordinate binding partner activities and impose hierarchies of interaction to control access to the processivity factor and hence to DNA. Further modulating interfaces have been proposed to assist in this with genetic, biochemical and structural characterisation clearly demonstrating that such interfaces play a crucial role [5,7,9,13,31,32]. However, a number of complexes, particularly those involving binding motifs from translesion polymerases, show subtle deviations in the mode of interaction that may impact on the polymerase usage hierarchy [7,12].

### The UmuC binding motif shows less reliance on the hydrophobic binding motif than related peptides

Given the agreement between the UmuC binding motif (QLNLF) and the consensus proposed by Dalrymple and others (2001) (QL[S/D]LF), it might be supposed that the UmuC motif would follow a very similar profile to that seen for Pol II (QLGLF) in complex with the β-clamp [14]. In the broadest context, the UmuC peptide binds to β-clamp as has been observed previously, occupying both binding sites on the clamp dimer, since crystal packing does not occlude either site. The principle clamp-binding peptide motif is ordered within the structure, with N-terminal elements disordered, as has been observed previously [11]. Little deviation is seen in binding at subsite 2, with the conserved glutamine making contact with the β-clamp backbone, both directly and via a conserved solvent molecule. The disposition of the two peptide leucine residues is again similar to the known structures, although UmuC Leu-360 is not inserted so deeply into the clamp hydrophobic pocket.

A dramatic and unique difference is seen in the conserved phenylalanine residue of UmuC. Insertion of the conserved phenylalanine into the clamp hydrophobic pocket at subsite 1 is accepted to be a crucial part of the β-clamp-binding partner interaction. Even where deviation has been observed previously, in the LGL motif of Pol IV, bending at the glycine allows the neighbouring leucines to come into close contact and they insert into the clamp hydrophobic pocket in a manner analogous to the phenylalanine in other structures. The conserved phenylalanine of the UmuC peptide does not insert into the clamp hydrophobic pocket at all, and this arrangement is reflected in the increasing deviation in Cα position moving through the peptide, as compared to the known complexes.

Mutational analysis of β-binding peptides typically emphasises the importance of the hydrophobic interaction between the peptide Leu-Phe motif and the clamp surface. Beuning and others (2006) assessed the relative mutational frequency of various UmuC mutants. Mutating UmuC Gln-357 to alanine reduced mutagenesis markedly, to ~20% WT levels. Interesting, mutating Asn-359 had a more profound impact than mutating Phe-361, reducing mutagenesis levels to ~30% and ~60%, respectively [33]. Mutating the equivalent glutamate (Glu-1156) in Pol III resulted in a clear reduction in β-binding, whilst mutation of Pol III Phe-1159 eliminated β-binding with the consequent effect that the phenylalanine mutant was incapable of competing Pol III core from the clamp, whilst the E1158A mutant was proficient [15]. Mutation of Pol III Phe-924 to alanine in the internal β-binding motif resulted in a 10-fold reduction in affinity compared to WT, although the comparable D922 mutation was not described in this study [20].

It appears that mutation of Phe-361 in UmuC has a less profound effect than equivalent mutations in either of the two Pol III β-binding motifs and, conversely, that mutation of Asn-359 in UmuC has a more pronounced effect than would be expected. We suggest that greater reliance is placed upon the UmuC Asn-359 to clamp Arg-152 interaction, since no intimate interaction exists between UmuC Phe-361 and the clamp, with Phe-361 involvement limited instead to interaction with the hydrophobic portion of the side chain of clamp Arg-246.

### Variations occur in the β-clamp binding motifs

Detailed bioinformatics analysis of β-clamp binding motifs suggests that the central residue in the motif is most likely to vary, with serine the most likely amino acid (34%), followed by aspartate (23%) with other small side chains frequently encountered [5]. Asn-359 in UmuC forms a hydrogen bond with clamp Arg-152. The nearest structural homologue, Pol II, possesses glycine at this location and a solvent molecule in the position comparable to the asparagine side chain. Pol III has glutamate in this position in the C-terminal β-motif; this side chain indirectly contacts clamp Arg-152 via water molecules, rather than forming a salt bridge, and instead interacts with the clamp via a hydrogen bond to His-175.

A number of mutant forms of the β-clamp have been identified that differentially affect the DNA polymerases of *E. coli*. β-clamp P363S and alanine mutants in the 173–175 region have been shown to have a more profound effect on Pol V function than on replication by Pol III [34]. UmuC Gln-357 forms a hydrogen bond with the carbonyl of clamp Pro-363 and the main chain amine group of UmuC Leu-360 interacts with the carbonyl of clamp Gly-174. The Pol III peptide shows the same interaction with clamp Pro-363, and a main chain interaction between Pol III Leu-1157 and clamp Gly-174 [14], suggesting that these interactions are more crucial to maintaining UmuC binding than Pol III.

It is clear from this study that, despite good agreement with the consensus sequence, variation in β-clamp binding is observed between the β-binding motifs of the *E. coli* polymerases. Although the peptides are not presented in the context of full length proteins which has been postulated to affect binding [11], it seems likely that these subtle variations in binding affect polymerase usage hierarchy and are influenced by key residues on the clamp surface. Detailed inspection of the complex structures shows that Arg-152 and His-175 on the clamp surface are particularly variable in their position. Arg-152, in particular, varies in terms of interaction with the peptides. Arg-246 also varies in position and interaction, though to a lesser extent.

### Interaction with Arg-152 and His-175 is affected by deviations in the binding motif

The central residues of the β-binding motif and the motif location on the protein, i.e. internal or C-terminal, appear to be the principal determinants of the position of both His-175 and Arg-152 in the clamp. For example, clamp His-175 is altered in complex with Pol III due to contact with Pol III Glu-1154, with only water-mediated interactions between Pol III residues and clamp Arg-152. In UmuC, Asn-359 is physically much closer to clamp Arg-152 due to the increasing deviation in position of the peptide backbone, and may indeed influence this deviation. The terminal oxygen of Pol II forms a water mediated interaction with clamp Arg-152, as does the terminal oxygen of Pol IV, but the six residue clamp-binding motif of Pol IV results in a greater shift in the clamp Arg-152 position.

Biochemical and genetic analyses have highlighted His-175 and Arg-152 as playing an important role in β-clamp function in the cell. Both residues exist in surface exposed loops and alanine mutants of clamp residues 173–175, also incorporating the key Gly-174 residue, and 148–152 have given insight into polymerase usage hierarchies [31,34]. The β-clamp 148–152 loop was shown to be critically important for Pol V function *in vivo*, as well as clamp Pro-363 as mentioned above. Individual clamp mutants including Arg-152 were analysed, but were thought to produce changes insufficient to interfere with mutagenesis *in vivo*[34]. Comparison of the single G174A and 173–175 clamp mutants, which result in distinct UV-sensitive phenotypes, suggested differential effects on the polymerases and it was proposed that in these mutants, Pol IV could gain control of the replication fork ahead of Pol V, which likewise would replicate in preference to Pol II.

**Figure 7:**
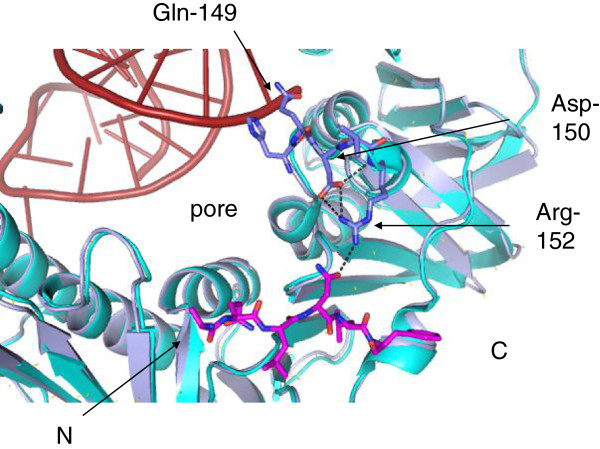
**The proposed communication relay between the β-clamp pore to the peptide binding site.** Gln-149 is proposed to act as a DNA sensor, impacting on the conformation of Asp-150, which in turn substantially interacts with Arg-152, a key clamp residue involved in peptide binding. The co-complex presented has been aligned with the β-clamp/DNA co-crystal structure [PDB:3BEP]. The β-clamps are shown in cartoon form in light blue (UmuC complex) and cyan (DNA complex), with the 148–152 loop shown in stick representation in atomic colouring. The UmuC peptide is shown in magenta, with key hydrogen bonds represented by black dashed lines. DNA is coloured red. The N- and C-terminal regions of the peptide are indicated.

Complicating matters further, clamp Arg-152 has been shown to contact the nascent DNA duplex when the β-clamp is bound to a primer-template terminus, with Gly-174 also making contact [3]. Neuwald proposed in 2003 that Gln-149 could function by sensing DNA within the pore of the clamp and relaying the information back to the peptide binding site [35] (Figure 7). A co-crystal structure of the β-clamp in complex with a primer-template terminus demonstrated interaction between Gln-149 and the oligonucleotide. The principle defect of Q149A in Pol III-mediated replication appeared to be at the clamp loading stage rather than elongation [3], also noted for the 148–152 clamp mutant [36], presumably due to a defect in DNA binding impacting on subsequent clamp loading at the primer terminus. In contrast the β-clamp 148–152 loop was shown to be crucial for both interaction with Pol II and IV and associated replication [36]. These results are consistent with the proposed relay of information upon DNA-binding to clamp Gln-149, via the conserved Asp-150 which hydrogen bonds extensively to Arg-152 [35]. D150N was isolated as a clamp mutant affecting action of the *umuDC* gene products [37]. Pol V is a classic example of the complexity of dissecting protein interactions and hence regulation; beyond the canonical binding motif described here, interactions have been characterised in the Pol V LF domain and sites at Arg-230, Thr-243 and within the CTD, at Leu-389 [33,38]. Additionally, the UmuD and UmuD’ proteins bind β-clamp directly and truncation of the UmuD protein to UmuD’ is itself crucial to regulation of Pol V activity [37].

### The UmuC binding motif is internal to the protein

One point of interest is that, unusually, although not uniquely, the UmuC clamp-binding motif is internal, lying between the LF domain and the CTD (the latter currently of unknown function). The UmuC peptide was designed to possess the two aspartate residues C-terminal to Phe-361, leading into the UmuC CTD. It seems likely that it is this arrangement which influences the unusual position of the UmuC Phe-361. The Pol III C-terminal peptide has one aspartate residue at the extreme C-terminus. In contrast to that seen in UmuC, for which no density was observed, the terminal residue of the Pol III peptide was well ordered, presumably a reflection of the intimate contact made between the clamp Arg-246 and the preceding phenylalanine in the peptide [14]. Presumably Asp-362 and Asp-363 do not form sufficiently stable contacts with the clamp surface for electron density to be observed. As has been observed previously, although many PIP box-containing proteins form a section of β-sheet with the interdomain connector loop of PCNA, this arrangement is precluded in the β-clamps by an insertion between the first helix and second strand in the second domain [7]. Therefore the presence of these residues is likely to be the main determinant for the lack of penetration of Phe-361 into the hydrophobic pocket.

It must also be considered that the UmuC CTD may contact the clamp, presumably in the region of domain 2. In the context of the full length protein this region may be less mobile in complex than observed in the peptide-protein co-crystal. The β-binding motif of the δ subunit of the clamp loader is internal and this results in the residues C-terminal to the conserved phenylalanine forming a turn back into the globular fold of the δ subunit [39]. In the two polymerases where the terminal residue of the clamp-binding motif is the extreme terminus of the protein (II and IV) the terminal oxygen groups both form water-mediated interactions with clamp Arg-152.

Interestingly, the other human translesion polymerase containing an internal PIP box, Pol ι, also shows deviation in the nature of its binding to the hydrophobic pocket of PCNA [12]. Pol ι possesses two tyrosine residues, with the unusual interaction between the hydroxyl group of Pol ι Tyr-427 and the carbonyl group of the lysine (412) substituting for the conserved glutamine in the peptide.

### Implications of variation

Clearly β-clamp surface residues affect the behaviour of the various clamp-binding peptide motifs, particularly Arg-152 and to a lesser extent, His-175. The subtle differences between these motifs are particularly relevant when considering the β-clamp as a potential antibiotic drug target. Full characterisation of key binding partners is critical to a successful outcome, since structural prediction is unlikely to highlight the type of differences observed in the crystal structure of the UmuC peptide with the β-clamp. Variation in binding between the polymerases has already been demonstrated to alter the efficacy of trial drugs, since RU7 is 50-fold less effective on Pol IV than Pol III, presumably reflecting the divergent Leu-Gly-Leu motif the former possesses over the canonical Leu-Phe motif of the latter [14]. The multiple peptide and drug complexes now available have highlighted clamp residues critical to binding that could be optimised for enhanced inhibitor binding, such as Arg-152 and Arg-246 [14,22] .

## Conclusion

Analysis of polymerase interaction with the β-clamp is complex, particularly when considering the multimeric Pol III and Pol V. It is increasingly apparent that subtle variation in the β-clamp binding motif in conjunction with modulating, non-overlapping interfaces of various binding partners plays a central role in determining this hierarchy and that direct binding of DNA by the clamp differentially influences polymerase binding. The structure presented here demonstrates the importance of characterisation of motifs at atomic level, despite their apparent agreement with consensus sequences. Asn-359 of UmuC plays an unexpectedly crucial role in β-clamp binding, owing to direct interactions with clamp residue Arg-152, with the conserved Phe-361 in UmuC playing a less significant role, presumably due to the context of the motif within the protein sequence. Understanding these processes is relevant to the characterisation of the fundamental process of DNA replication and dissection of the regulation of mutagenesis, impacting on the adaptation of bacterial pathogens, and the development of next generation antibiotics.

## Abbreviations

Pol: Polymerase; LF: Little finger (domain); CTD: C-terminal domain; PIP: PCNA-interacting peptide.

## Competing interests

The authors declare there are no competing interests.

## Authors’ contributions

AAP purified protein, produced material for crystallisation and performed crystallisation. JAW assisted with crystallisation and cryo-preservation. KAB designed the experiment and, with AAP, performed the structure solution. JAW and KAB wrote the manuscript. All authors read and approved the final manuscript.

## Supplementary Material

Additional file 1: Figure S1. Omit density maps showing the peptide (chain D) in stick representation. The surface of the β-clamp is shown in magenta, with the 2Fo-Fc density map in grey, contoured at 1σ and the Fo-Fc map in dark blue, contoured at 3 σ. **Figure S2.** Summary of key interactions between clamp-binding peptide motifs of *E. coli* polymerases and the β-clamp. Dashed lines show interactions (direct or solvent-mediated) discussed in this manuscript. Asterisks indicate residues forming the β-clamp hydrophobic binding pocket. (≡) denotes equivalent residue in the UmuC clamp-binding peptide for comparison. More detailed representation of interactions between UmuC and the β-clamp is shown in Figure 3. **Figure S3.** Superposition of the UmuC complex (magenta/light pink) with the uncomplexed β-clamp (orange). Met-362 is indicated with a red dotted ring. Click here for file
