# Supplementary material for: The UmuC subunit of the E. coli DNA polymerase V shows a unique interaction with the β-clamp processivity factor
Source: BMC Struct Biol. 2013 Jul 4;13:12. doi: 10.1186/1472-6807-13-12 (PMC3716654; doi:10.1186/1472-6807-13-12)

Supplementary Figure 1. Omit density maps showing the peptide (chain D) in stick representation. The surface of the β-clamp is shown in magenta, with the 2Fo-Fc density map in grey, contoured at 1σ and the Fo-Fc map in dark blue, contoured at 3 σ.

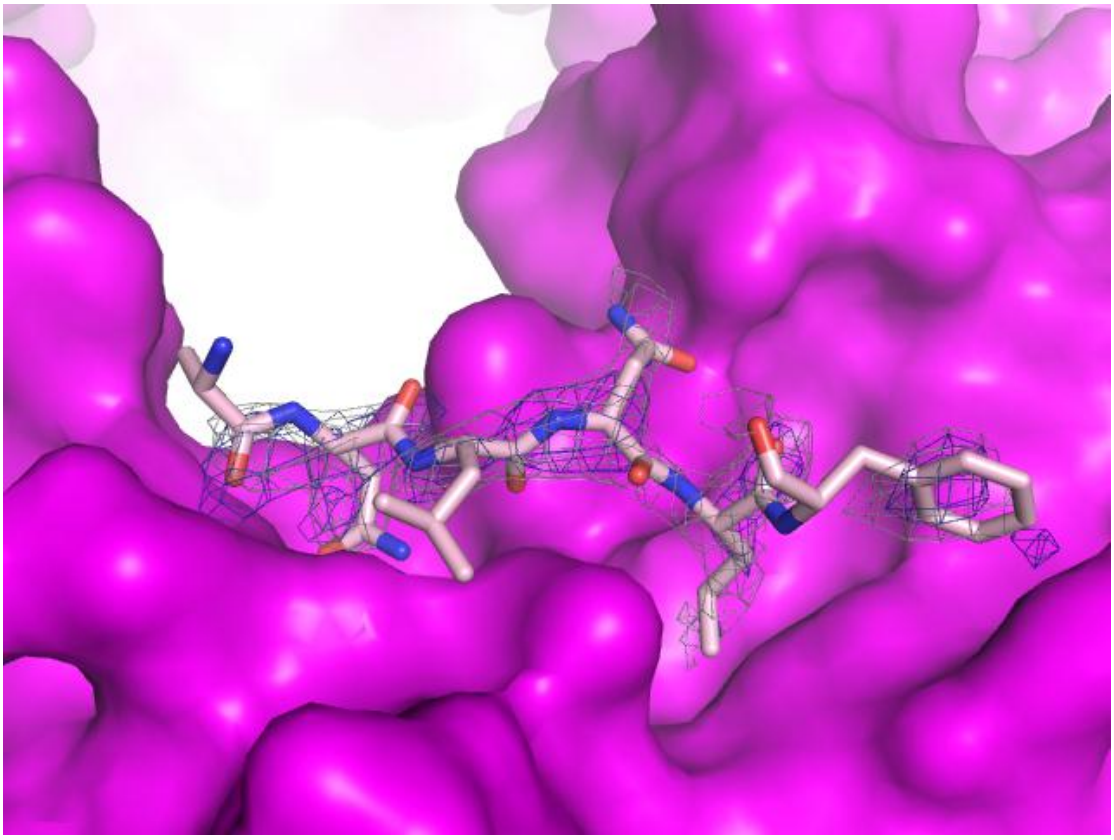

Supplementary Figure 2. Summary of key interactions between clamp-binding peptide motifs of *E. coli* polymerases and the β-clamp. Dashed lines show interactions (direct or solvent-mediated) discussed in this manuscript. Asterisks indicate residues forming the β-clamp hydrophobic binding pocket. (≡) denotes equivalent residue in the UmuC clamp-binding peptide for comparison. More detailed representation of interactions between UmuC and the β-clamp is shown in Figure 3.

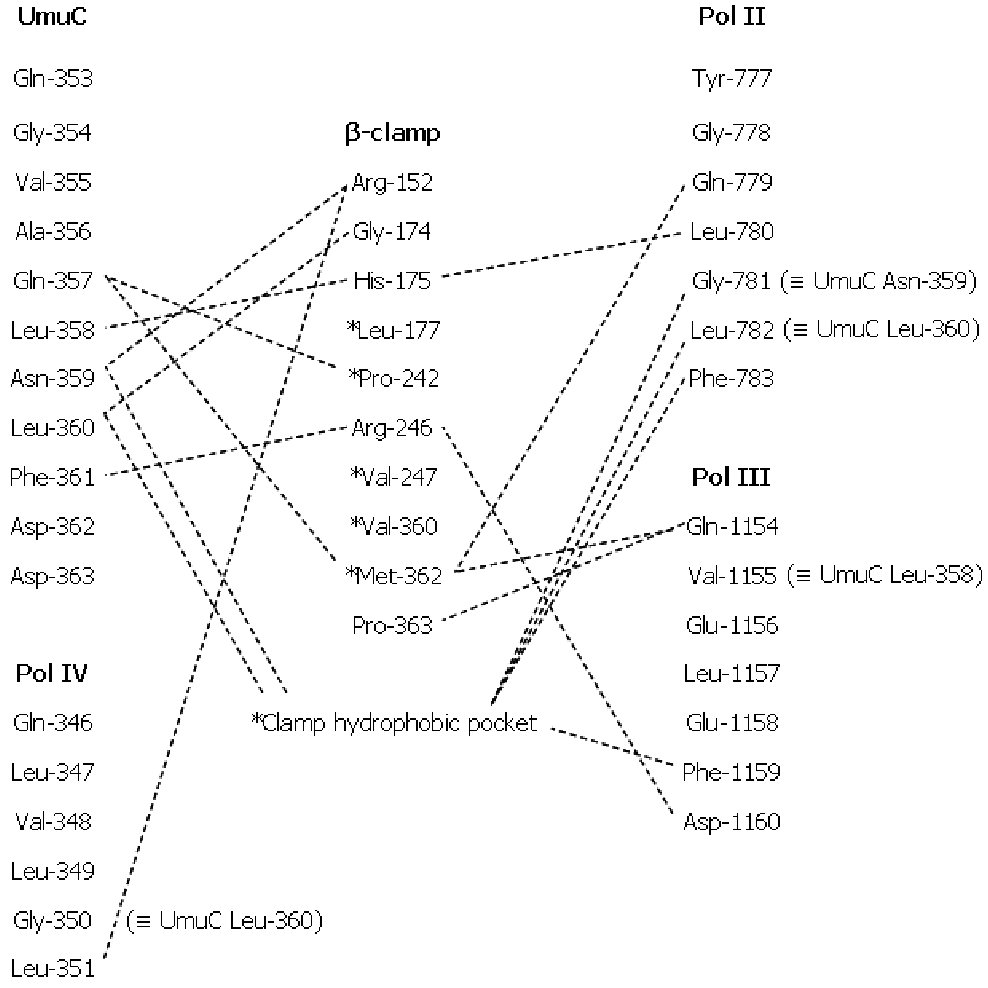

Supplementary Figure 3. Superposition of the UmuC complex (magenta/light pink) with the uncomplexed β-clamp (orange). Met-362 is indicated with a red dotted ring.

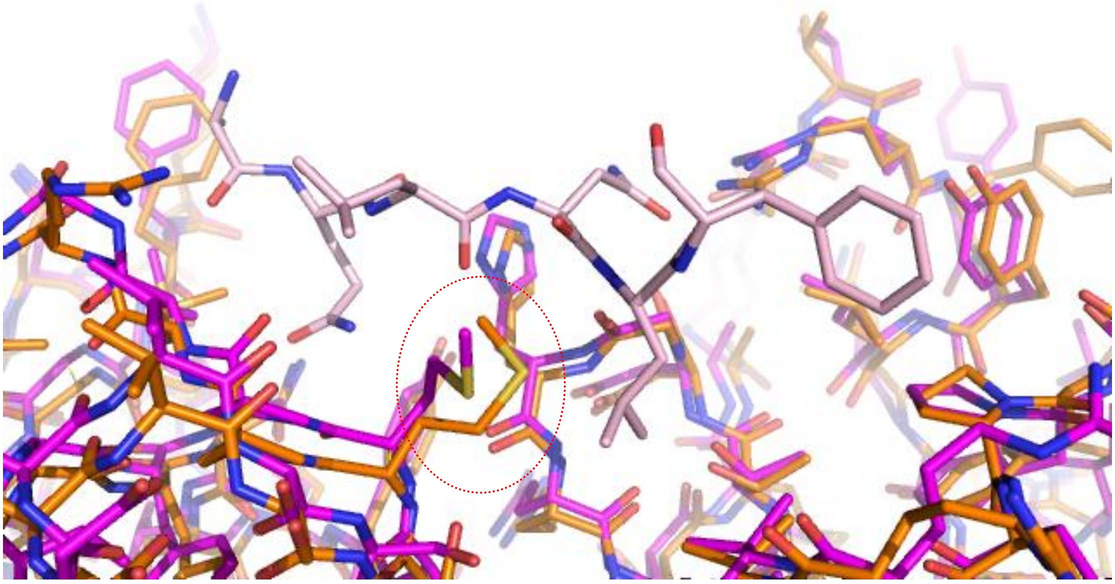

Supplement: Additional file 1: Figure S1. — Omit density maps showing the peptide (chain D) in stick representation. The surface of the β-clamp is shown in magenta, with the 2Fo-Fc density map in grey, contoured at 1σ and the Fo-Fc map in dark blue, contoured at 3 σ. Figure S2. Summary of key interactions between clamp-binding peptide motifs of E. coli polymerases and the β-clamp. Dashed lines show interactions (direct or solvent-mediated) discussed in this manuscript. Asterisks indicate residues forming the β-clamp hydrophobic binding pocket. (≡) denotes equivalent residue in the UmuC clamp-binding peptide for comparison. More detailed representation of interactions between UmuC and the β-clamp is shown in Figure 3. Figure S3. Superposition of the UmuC complex (magenta/light pink) with the uncomplexed β-clamp (orange). Met-362 is indicated with a red dotted ring. [file 1472-6807-13-12-S1.pdf]
